# Supplementary material for: The Role of Bone Marrow Stromal Cell Antigen 2 (BST2) in the Migration of Dendritic Cells to Lymph Nodes
Source: Int J Mol Sci. 2024 Dec 27;26(1):149. doi: 10.3390/ijms26010149 (PMC11720714; doi:10.3390/ijms26010149)
Supplement: Supplementary file 1 [file ijms-26-00149-s001.zip › ijms-3303381-supplementary.pdf]

International Journal of
Molecular Sciences

MDPI

Supplementary data

The Role of Bone Marrow Stromal Cell Antigen 2 (BST2) in the Migration of Dendritic Cells to Lymph Nodes

Sehoon Park †, Eunbi Yi †, Jaemyeong Jeon, Jinsoo Oh, Zhengmei Xu and Se-Ho Park *

College of Life Sciences and Biotechnology, Korea University, 145 Anam-ro, Seongbuk-gu, Seoul 02841, Republic of Korea; shoon153@naver.com (S.P.); pinelime@naver.com (E.Y.); woaudasd@korea.ac.kr (J.J.); oh_jinsoo@korea.ac.kr (J.O.); jungmi69512@naver.com (Z.X.)

* Correspondence: sehopark@korea.ac.kr; Tel.: +82-2-3290-3160

† These authors contributed equally to this work.

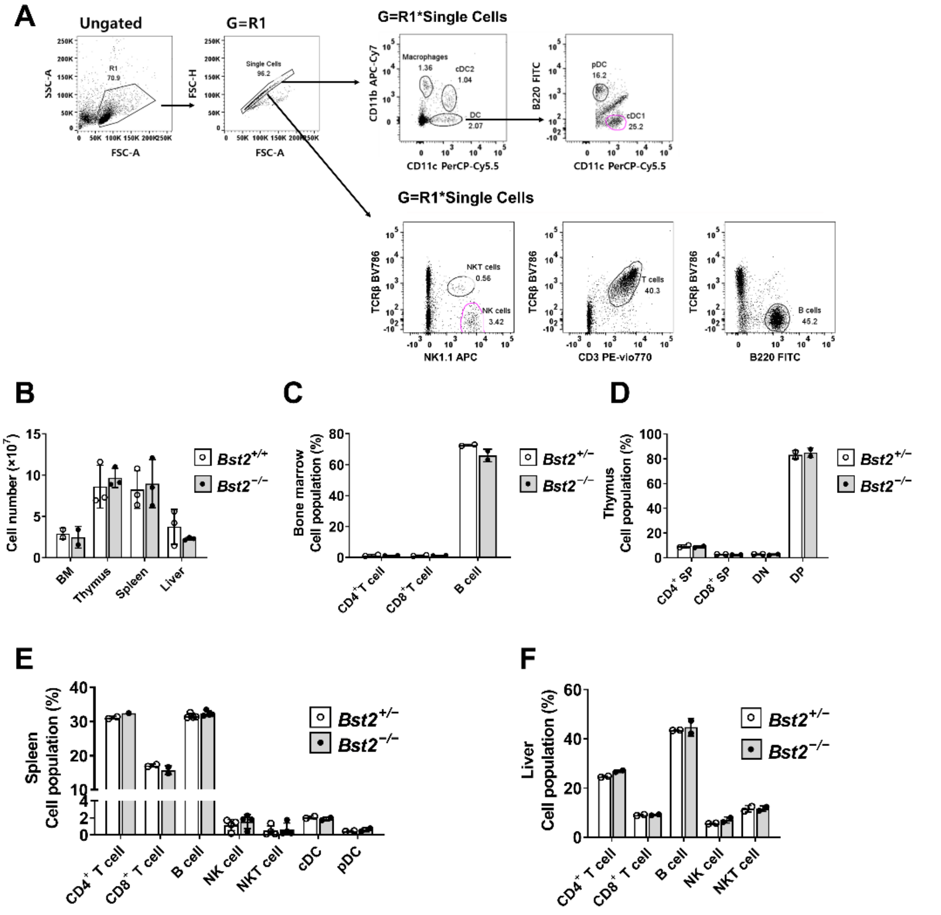

Supplementary Figure S1. *Bst2*$^{-/-}$ mice have normal cellularity and distribution of immune cells. (A) An example of gating strategy for cell populations in the splenocytes. (B) The absolute cell numbers (cellularity) isolated from the bone marrow, thymus, spleen, and liver in *Bst2*$^{+/+}$ and *Bst2*$^{-/-}$ mice were counted. (C-F) To investigate the distribution of immune cell subsets, cell suspensions isolated from each organ were stained using fluorochrome-conjugated cell surface marker-specific antibodies. CD4+ T cells, TCRβ+CD4+; CD8+ T cells, TCRβ+CD8+; B cells, TCRβ-B220+; NK cells, NK1.1+TCRβ-; NKT cells, NK1.1+TCRβ+; Conventional dendritic cells (cDCs),

CD11b+CD11chigh; Plasmacytoid dendritic cells (pDCs), CD11b-CD11clowB220+. Representative data are mean ± S.D. from three independent experiments with similar results.

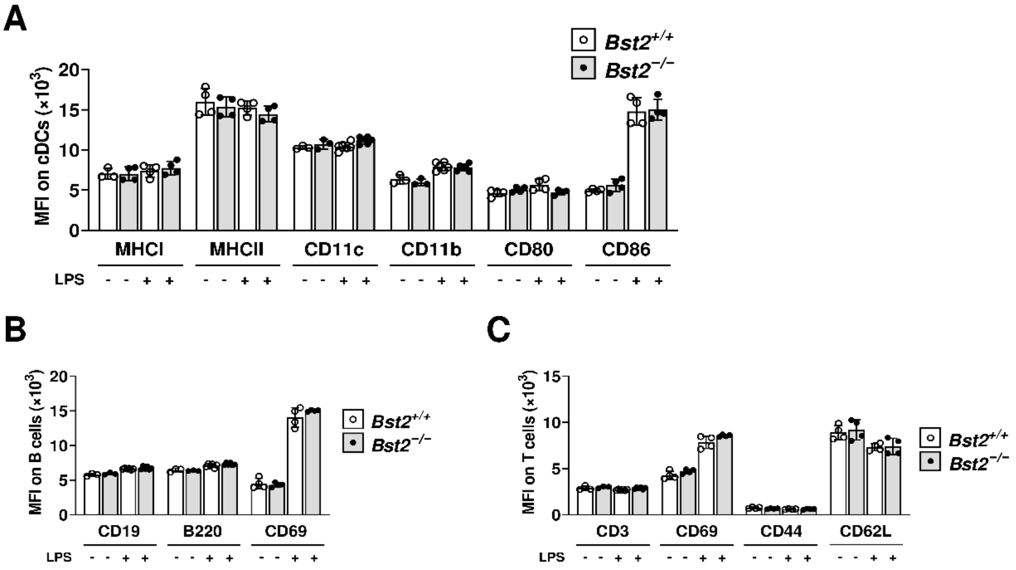

Supplementary Figure S2. *Bst2*$^{-/-}$ mice have normal expression of cell surface molecules. Splenocytes derived from *Bst2*$^{+/+}$ and *Bst2*$^{-/-}$ mice were unstimulated or stimulated with 0.1 μg/ml of LPS for 24 hours and mean fluorescence intensity (MFI) of lineage markers on (A) cDCs, (B) B cells and (C) T cells were determined by flowcytometry analysis. Representative data are shown as mean ± S.D. from two independent experiments with similar results.

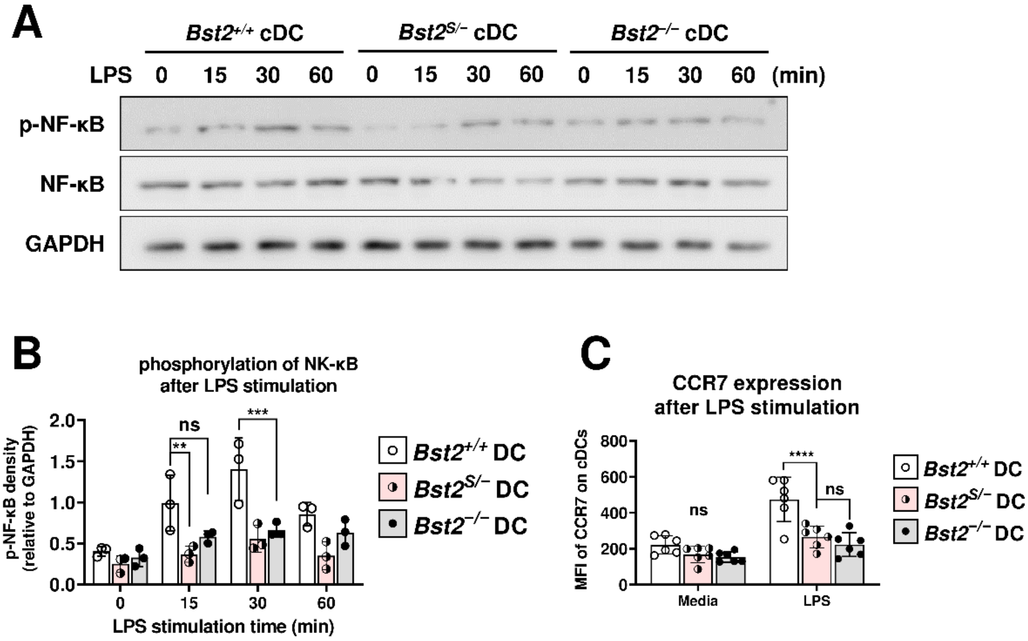

Supplementary Figure S3. NF-κB-mediated CCR7 expression is involved in BST2. Sorted *Bst2*$^{+/+}$, *Bst2*$^{S/-}$ (short BST2) and *Bst2*$^{-/-}$ cDCs were stimulated with 1 μg/ml of LPS for indicated times. (A) The expressions of NF-κB/p65 and phosphorylated NF-κB/p-p65 in the cytosolic fraction were detected by Western blotting. GAPDH was used as a loading control. (B) The corresponding densitometric analysis of all Western blot results was normalized to GAPDH. (C) *Bst2*$^{+/+}$, *Bst2*$^{S/-}$ and *Bst2*$^{-/-}$ mice-derived splenocytes were unstimulated or stimulated with 1 μg/ml of LPS for an hour

and mean fluorescence intensity (MFI) of CCR7 was measured by flowcytometry analysis. Representative data are shown as mean ± S.D. from three independent experiments with similar results.
